# Case Study on Prestressed CFRP Plates Applied for Strengthening Hollow-Section Beam Removed from an Old Bridge

**DOI:** 10.3390/polym15030549

**Published:** 2023-01-20

**Authors:** Guirong Liu, Bingchen Li, Jiasheng Bao, Shengzhao Cheng, Qingxin Meng, Shunbo Zhao

**Affiliations:** 1 International Joint Research Lab for Eco-Building Materials and Engineering of Henan, North China University of Water Resources and Electric Power, Zhengzhou 450045, China; 2 China Construction Seventh Engineering Bureau Co., Ltd., Zhengzhou 450004, China; 3 Collaborative Innovation Center for Efficient Utilization of Water Resources, North China University of Water Resources and Electric Power, Zhengzhou 450046, China

**Keywords:** full-scale existed concrete beam, CFRP plate, prestressing technology, experiment study, finite element model, parametric analysis, loading performance

## Abstract

With the wide application of carbon fiber reinforced polymer (CFRP) plate, used for strengthening existed concrete structures, the prestressing technology of CFRP plate is becoming a hot topic, in order to sufficiently develop its high-strength peculiarity. In this paper, a full-scale hollow-section beam with length of 16 m taken from an old bridge which was in service for about 20 years was first examined for existed cracks and repaired by filling epoxy adhesive, and then the beam was strengthened with prestressed CFRP plates. The CFRP plates were tensioned and fixed with flat-plate anchorages at ends and bonded with adhesive on the bottom surface of the beam. The strengthened beam was experimentally studied using a four-point test to measure the concrete strain along the height of the mid-span section and the mid-span deflection. The finite element model of the strengthened beam was verified by the comparison of test results and used for an extending study of parametric analysis considering the effect of the length and amount of CFRP plates. Results indicated that with an increase in the length and amount of CFRP plates, the mid-span deflection of the beam decreases with the increased cracking resistance and bearing capacity, while the ultimate failure mode transfers from the under-reinforcement to the over-reinforcement.

## 1. Introduction

Prestressed concrete hollow-plate bridges are commonly constructed in highway and railway engineering with the advantage, including relatively lightened self-weight, of fast construction using cast-in-situ concrete with assembling of precast members [1,2,3]. In the service progress of a bridge, several reasons, including the environment effect, inadequate maintenance, and excessive vehicle load, will lead to the performance degradation of bridge structure in bearing capacity and durability [3,4,5]. To ensure traffic flow and ease the pressure on reconstruction funds, the worsened bridges are usually strengthened instead of rebuilt [6,7,8].

Regarding the strengthening methods for concrete structures, strengthening with fiber reinforced polymer (FRP) composites including carbon FRP, glass FRP and hybrid FRP, is a well-accepted technique due to its lightweight, high-strength, and good corrosion resistance [9,10,11,12]. Normally, the externally bonded method is adopted with longitudinal FRP laminates on the bottom surface to strengthen the flexural capacity, or with FRP U-jackets on vertical sections to strengthen the shear capacity of reinforced concrete beams [13,14,15]. In this aspect, the debonding failure rather than the fracture of FRP leaves the issue of insufficient utilization of the high strength of FRP [16,17,18,19]. Therefore, a lot of research attempts to employ appropriate techniques to avoid FRP debonding. Among them, a method of near-surface-mounted FRP strips, via cutting a groove into the concrete surface and filling the FRP strips and epoxy paste, can improve the bearing capacity of strengthened reinforced concrete beams compared with those of externally bonded FRP when the reinforcement amount is equivalent [20,21,22,23]. Meanwhile, the anchoring methods for FRP laminate/sheets with mechanically fastened anchors which prevent the debonding of externally bonded CFRP can effectively delay debonding and enhance the deformability as well as the bearing capacity of strengthened and reinforced concrete elements [24,25,26,27].

In order to develop the advantage of high-strength with a large stiffness of carbon FRP (CFRP), the prestressing method has been created to increase the strength utilizing efficiency of CFRP with a rational economy for improving the strengthening effect on reinforced concrete beams [9,28,29,30,31]. This can be through strengthening the reinforced concrete beams by CFRP plates at different prestress level to let the strengthened beams reach the bearing capacity with the fracture of CFRP plate at a high prestress level. In recent years, Yang et al. [32] proposed a self-anchored method of prestressed CFRP plates; the results of the experimental study indicated that the flexural stiffness and bearing capacity of the strengthened reinforced concrete beams were significantly improved with comparison to those strengthened by external bonded CFRP. A total of 81% tensile strength of the prestressed CFRP plate could be utilized, although the debonding of CFRP plate was not completely avoided. Xie et al. [33] conducted an experimental study on the reliability of prestressed CFRP plates in anchor systems, assessing the effects of existing damage in reinforced concrete beams before strengthening and overloading after strengthening. Results indicated that the coupling action of corrosion- and cyclic overload-induced damage remarkably reduced the flexural stiffness but did not greatly impact upon the ultimate load of the strengthened beams. After exposure to cyclic overloading, the strengthened beams had an ultimate load retention of more than 80% and had a similar failure mode of concrete crushing. Another method of strengthening reinforced concrete beams using prestressed CFRP plates, proposed by Yang et al. [34], was that the CFRP plate was fixed by corrugated anchors at ends of beam and stressed by adjusting the position of the mid-span deviators. Experimental results showed that the maximum stress of the CFRP plate reached 1500 MPa which was about half of the tensile strength of the CFRP plate, and the beam strength was enhanced by 43%, while some of the strengthened beams achieved good ductility.

Moreover, experimental research on full-scale reinforced concrete beams strengthened with prestressed CFRP plates have been performed. Wang et al. [35] conducted an experimental study to assess the effects of the prestressing level, the strengthening amount, the type and the bonding position of the prestressed CFRP plate on the flexural behavior of large-scale strengthened reinforced concrete beams. Results showed that the post-cracking stiffness, the cracking and the yielding loads increased by increasing the prestressing level and the strengthening amount of the prestressed CFRP plate, and a high prestressing level of 50% could lead to a premature intermediate debonding of the CFRP plate before the yielding of the steel rebars, while the prestressed CFRP plate bonded on the two sides provided a lower flexural strengthening efficacy than that bonded on the bottom of the beam, owing to the shorter force arm and the nonuniform stress of the side-bonded CFRP plate. Li et al. [36] studied the flexural behavior of full-scale and damaged reinforced concrete beams strengthened with prestressed steel-carbon fiber reinforced polymer (SCFRP). Results showed that the stiffness at the elastic stage was increased by 64.9–67.1% after being strengthened by SCFRP with a 30–60% prestressing level, and the ultimate load were improved by 19.53–31.9%. The flexural behavior of the severely damaged reinforced concrete beam with a strength reduction coefficient of 0.65 can be recovered after being strengthened by SCFRP with 40% prestressing levels. These studies indicate that the prestressing technique of CFRP is effective in the strengthening of concrete structures.

To enrich research on the loading behaviors of full-scale reinforced concrete members strengthened with pressed CFRP plates, a full-scale hollow-section prestressed concrete beam was removed from an old bridge in operation for about 25 years, and transported to the lab in Zhengzhou, China, about 150 km from in-situ. The status of the beam was firstly detected to determine the properties of the concrete and the bearing performance of the hollow beam, and then the cracks were repaired by filling the epoxy paste to ensure the entirety of the beam. After that, the beam was strengthened by using prestressed CFRP plates, and the loading performance of the strengthened beam was experimentally studied by a four-point test. With the experience of using finite element (FE) analysis for the simulation of the loading process on reinforced concrete structures [37,38], the FE models of the proto and strengthened beams were built, and the test results were verified by the comparation with the numerical results of FE models.

Due to the limits of the experimental study, an extending parametric study was conducted to get a wide applicability of the experimental results. The effects of the length and the amount of prestressed CFRP plates on the flexural properties of prestressed concrete beams were analyzed using the FE model. Results provided a scientific basis for the strengthening technology of prestressed CFRP plates when applied to improve the loading capacity of old bridges, and the results could also be beneficial for similar projects.

## 2. Status of Full-Scale Hollow-Section Beam Removed from Old Bridge

### 2.1. Dimension and Reinforcements of the Proto Beam

**Figure 1:**
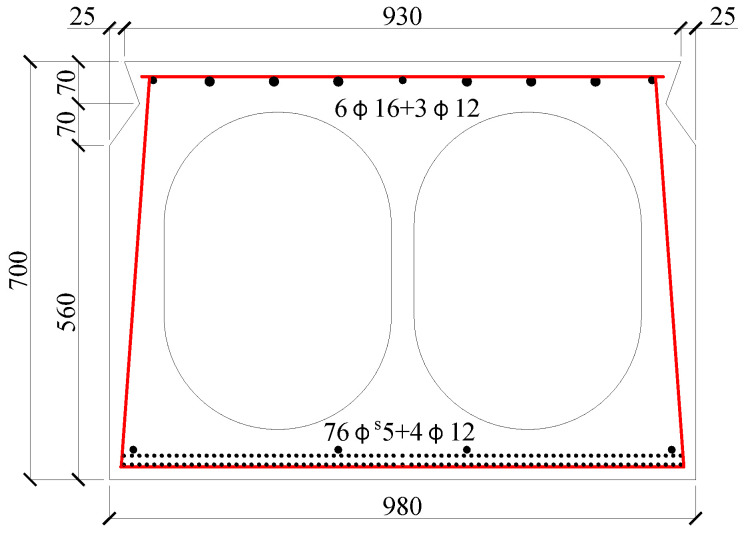
The geometry and size of the hollow-section beam (mm).

The prestressed concrete hollow-section beam investigated in this study was removed from an old simple-support assembly precast prestressed concrete bridge. This bridge was designed in accordance with China code JTG D62-85 [39], completed and formally operated in 1987. The design load was class 20-automobile and class 100-trailer. During its service of about 25 years, the design code was renewed twice as JTG D62-2004 and JTG 3362 [40,41]. However, the bridge does not meet the safety requirement of the current code, and need to be repaired and strengthening by rational technology. Figure 1 presents the geometry of the cross-section and the detailed reinforcement. The nominal width of the cross-section was 1000 mm, and the depth was 700 mm. The length of the beam was 16 m, and the span was 15.5 m. The longitudinal tensile reinforcement was 76ф^s^5 prestressed steel wire. A total of 4ф12 plane hot-rolled steel bars were placed in the tensile zone, 6ф16 + 3ф12 plane hot-rolled steel bars were placed in the compressive zone, and ф6 plane hot-rolled steel stirrups in spacing from 150 mm to 200 mm were used to form a reinforcement skeleton. The concrete cover of the outside reinforcements was 28 mm.

### 2.2. Status Detection for the Proto Beam

**Figure 2:**
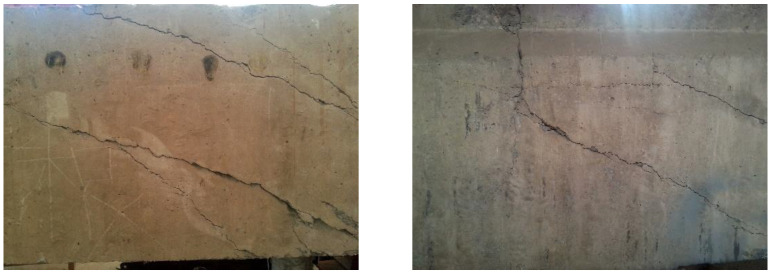
Cracks appeared on the proto beam.

Due to the long-term service of this old bridge, several cracks appeared on the side and bottom surfaces of the proto beam, as shown in Figure 2.

According to the specification of China code JGJ/T 23, the rebound method was used to inspect the concrete compressive strength [42]. Ten zones without cracks on the surface of the proto beams were selected to complete the rebound test. The cubic compressive strength of concrete was determined as 36.9 MPa. Based on the specification of China code GB 50010 [43], the prism axial compressive strength of concrete was 24.7 MPa, the tensile strength was 1.60 MPa, and the modulus of elasticity was 3.21 × 10^4^ MPa.

After the test of the strengthened beam, samples of prestressed steel wire were taken from the ends of the beam to measure the mechanical properties according to China code JGJ/T 152 [44]. Results indicated that the tensile strength and the modulus of elasticity of the steel wire were 1600 MPa and 1.95 × 10^5^ MPa.

### 2.3. Repair of Concrete Cracks

**Figure 3:**
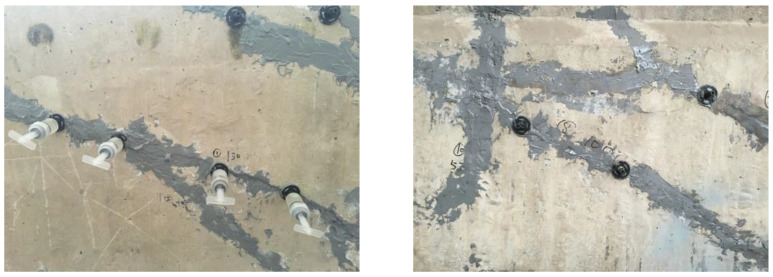
Repair of concrete cracks by filling epoxy paste.

To maintain the integrity of the proto beam, the existed cracks should be repaired before strengthening. Based on the specification of China code GB 50367 [9], the epoxy adhesive was used to fill the cracks, as presented in Figure 3. The adhesive condition was detected by the impact echo method specified in China code JGJ/T 411 [45]. The repair quality met the requirement of concrete integrity.

### 2.4. Loading Test of the Repaired Beam

**Figure 4:**
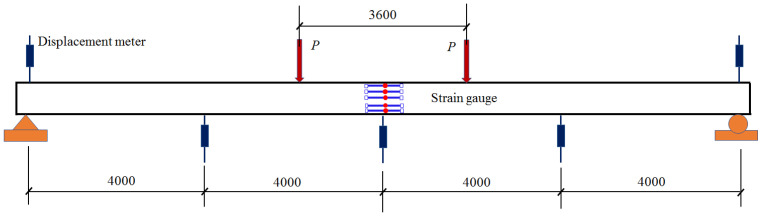
Arrangement of the repaired beam under four-point bending test.

Although the existing cracks were repaired by filling epoxy adhesive, it was hard to avoid the stiffness degradation of the proto beam. To evaluate the degree of stiffness degradation, a four-point bending test with a pure bending length of 3.6 m was conducted, as presented in Figure 4. The deflection of the repaired beam was measured by the displacement meters installed at one-fourth of the span, mid-span and the supports. The concrete strains at the mid-span section were measured by the strain gauges longitudinally installed along depth of the section. Test data were collected by an automatic data acquisition instrument.

**Figure 5:**
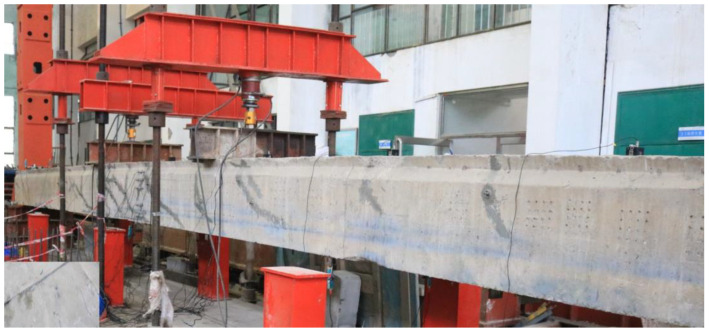
Photo of the repaired beam under four-point bending test.

Two concentrated loads were applied step by step on the top surface of the repaired beam. As shown in Figure 5, each load was exerted by a hydraulic jack, and controlled by a load meter.

Based on the specification of China code JTG 3362 [41], the maximum transverse distribution coefficient of moving vehicles on the old bridge was 0.238. At the serviceability limit state and the bearing capacity limit state, the target moment at the mid-span section were 892.0 kN·m and 955.0 kN·m, respectively. Therefore, the corresponding applied moments were 653.5 kN·m and 716.5 kN·m, calculated by subtracting the bending moment caused by self-weight.

In this study, the target load was determined with the bending moment at the serviceability limit state. Therefore, each concentrated load on the top surface of the repaired beam was 109.8 kN, denoted by *P*_0_. The loading step followed as 0.2, 0.4, 0.6, 0.8, 0.85, 0.9, 0.95, and 1.0 times the target load *P*_0_.

The stiffness degradation of the repaired beam was quantified by the following method. The stiffness *B*_ss_ of the repaired beam was obtained by the formula based on the test mid-span deflection *a*_f_,

(1) $$B_{ss}=\frac{\alpha P_{0}l^{3}}{a_{f}}$$

where *α* is coefficient, the value of *α* is 0.038 in this test; *l* is the span of the repaired beam, taken as 15.6 m; and *a*_f_ is the mid-span deflection at serviceability limit state.

The stiffness *B*_s0_ of the proto beam without degradation was calculated according to the formula specified in China code GB 50010 [43],

(2) $$B_{s0}=\frac{E_{s}A_{s}h_{0}^{2}}{1.15\psi +0.2+6\alpha_{E}\rho }$$

(3) $$\psi =1.1-\frac{0.65f_{\mathrm{tk}}}{\rho_{\mathrm{te}}\sigma_{\mathrm{sq}}}$$

where *E*_s_ is the modulus of elasticity of the longitudinal tensile steel bars; *α*_E_ is a ratio of the modulus of elasticity for steel bar to concrete; *ρ* is the reinforcement ratio of longitudinal tensile steel bars; *f*_tk_ is the tensile strength of concrete; *ρ*_te_ is the reinforcement ratio of longitudinal tensile steel bars in an effective sectional area; and *σ*_sq_ is the tensile stress of longitudinal tensile steel bars across cracks under the service load.

Therefore, the stiffness degradation coefficient *β*_f_ was obtained as follow,

(4) $$\beta_{f}=\frac{B_{\mathrm{ss}}}{B_{s0}}$$

Based on the test, the mid-span deflection was 54.1 mm at the serviceability limit state. Substituting the values of other parameters into above formulas, it was calculated that *β*_f_ = 0.73. This degradation was applied to the FE model of the repaired and strengthened beams by reducing the tensile strength of concrete in the pure bending segment. That is, the tensile strength of concrete was taken as 0.73 times 1.60 MPa as 1.17 MPa.

## 3. Strengthening Method and Test of Strengthened Beam

### 3.1. Strengthening Method of Prestressed CFRP Plates

The prestressing of CFRP plates was used to strengthen the repaired beam. The design method specified in China code GB 50367 was applied to determine the amount of CFRP plates [9]. Based on the design principle of ensuring under-reinforced failure mode for the repaired beam after strengthening, three prestressed CFRP plates were used in this study. Each plate was 54 mm wide, 1.4 mm thick, and 10 m long. The tensile strength and the modulus of elasticity of prestressed CFRP plate were 2400 MPa and 1.60 × 10^5^ MPa, respectively. The tensioning stress was 720 MPa, 30% of the ultimate tensile strength of the CFRP plate. The effective prestress was 533.3 MPa which determined by deducting the prestress losses due to the anchor deformation and locking shrinkage, and the relaxation of CFRP plate. Therefore, according to the formula specified in China code GB 50367 [9], the bending moments at mid-span were increased by 79.0 kN·m and 167.0 kN·m at concrete cracking and ultimate bearing conditions, respectively.

**Figure 6:**
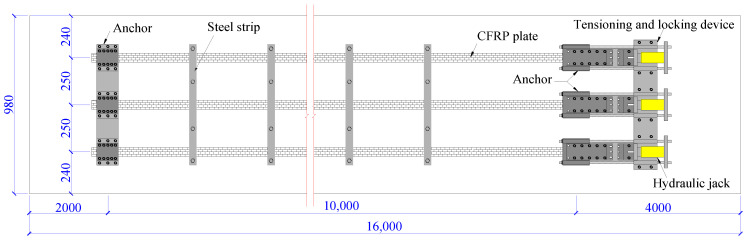
Schematic diagram of the prestressing of CFRP plates.

**Figure 7:**
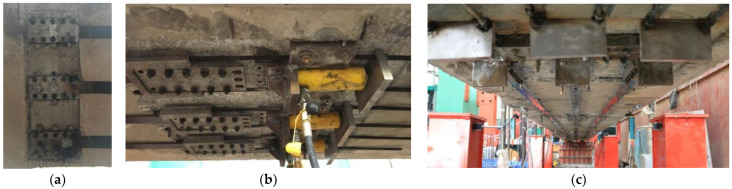
Photos of strengthened beam: (**a**) fixed end; (**b**) tensioning end; (**c**) strengthening completed.

As shown in Figure 6, the prestressing system consists of the anchors at fix and tensioning ends, the prestressed CFRP plates, the steel strips, and the tensioning and locking devices. The flat anchor is composited by two opposite steel plates with inner surface indentations, steel screws, bolt, and nut, which acts as the anchorage after the tensioning of prestressed CFRP plates [46]. The anchors at the fixed end and the tensioning and locking devices were installed on the bottom surface of the repaired beam by chemical bolts with adhesive. After tensioning the CFRP plates by hydraulic jacks, the anchors at tensioning end were firstly locked by the locking rods, and then fixed by chemical bolts with adhesive. When the adhesive hardened with efficient strength, after about 24 h, the locking rods were relaxed, and the tensioning and locking devices were removed before the loading test. This made the repaired beam a strengthened beam, as shown in Figure 7.

### 3.2. Loading Test of the Strengthened Beam

The strengthened beam was tested using the same loading method as presented in Figure 4. To investigate the reinforcement effect of prestressed CFRP plates, the load was firstly added step by step to the service load *P*_0_. After that, the load was exerted until the strengthened beam failed. The value of the targeted ultimate bending moment was that of the repaired beam plus the strengthened by prestressed CFRP plates, that is, 716.5 + 167.0= 883.5 kN·m.

## 4. FE Models of Proto and Strengthened Beams

### 4.1. Elements and FE Meshes

**Figure 8:**
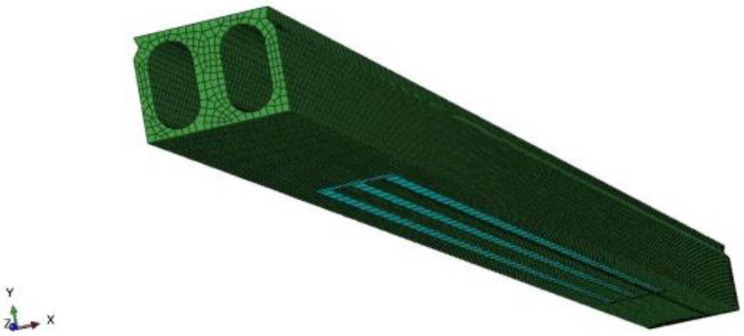
FE model of the strengthened beam.

Nonlinear FE analysis of the repaired beam and the strengthened beam were made by using the ABQUAS software. Figure 8 shows the FE model of the strengthened beam.

The C3D8R element was used for concrete, which was a eight-node linear block with reduced integration. The S4R element was used for the CFRP plates, which was a four-node doubly curved thin shell with reduced integration. The T3D2 element was used for the steel bars, and the nonlinear spring element Spring2 was used to simulate the bond-slip between prestressed steel wire and concrete. The cohesive contact was used to simulate the interfaces between CFRP plates and concrete. In view of the sectional dimension of the beam, most of the concrete elements were divided in sizes of 100 mm. Those with the size of 50 mm were also used to simulate the hollow to reduce stress concentration.

### 4.2. Modeling of Materials

The concrete damage plasticity (CDP) model based on Lubliner et al. [47], Lee and Fenves [48] was selected in this study. The degradations of the tensile and compressive elastic modulus of concrete were described by the tension and compression stiffness damage factors. The formulas of the constitutive elements of concrete are written as follows.

Concrete tension:

(5) $$\sigma =(1-d_{t})E_{c}\varepsilon$$

(6) $$d_{t}=\left\{ \begin{array}{ll} 1-\rho_{t}\left[1.2-0.2x^{5}\right] & \;\;x\leq 1\\ 1-\frac{\rho_{t}}{\alpha_{t}{\left(x-1\right)}^{1.7}+x} & \;\;x>1 \end{array}\right.$$

(7) $$x=\frac{\varepsilon }{\varepsilon_{t,r}},\;\;\rho_{t}=\frac{f_{t,r}}{E_{c}\varepsilon_{t,r}}$$

Concrete compression:

(8) $$\sigma =(1-d_{c})E_{c}\varepsilon$$

(9) $$d_{c}=\left\{ \begin{array}{ll} 1-\frac{\rho_{c}n}{n-1+x^{n}} & \;\;x\leq 1\\ 1-\frac{\rho_{c}}{\alpha_{c}{\left(x-1\right)}^{2}+x} & \;\;x>1 \end{array}\right.$$

(10) $$x=\frac{\varepsilon }{\varepsilon_{c,r}},\;\rho_{c}=\frac{f_{c,r}}{E_{c}\varepsilon_{c,r}},\;n=\frac{E_{c}\varepsilon_{c,r}}{E_{c}\varepsilon_{c,r}-f_{c,r}}$$

where *d*_t_ and *d*_c_ are the tension and compression damage factors of concrete, respectively; *E*_c_ is the modulus of elasticity of concrete; *f*_t,r_ and *f*_c,r_ are the peak tensile and compressive stress, respectively; and *ε*_t,r_ and *ε*_c,r_ are the tensile and compressive strain corresponded to the peak stresses, respectively.

Prestressed steel wire was assumed to be a linear elastic material. CFRP plate was assumed to be an anisotropic material which can be specified in the model by “Lamina” material type [49,50]. The main parameters were elastic modulus in direction of fibers and perpendicular to the fiber direction *E*_1_, *E*_2_, the rigidity module *G*_12_, *G*_13_, *G*_23_, and the Poisson coefficient *N*_u12_. Test values of these parameters are presented in Table 1.

### 4.3. Bond-Slip Relationships

The interface between prestressed steel wire and concrete was nodal, connected using a tied contact element Spring2 [51]. The bond-slip constitutive is expressed as:

(11) $$\frac{\tau_{f}}{\sqrt{f_{c}^{\prime }}}=\left\{ \begin{array}{ll} 0.41+0.11\sqrt{1-\frac{{\left(s-0.01\right)}^{2}}{0.01^{2}}}\; & \;0\leq s\leq 0.01\mathrm{mm}\;\\ 0.52-0.26\left(s-0.01\right) & \;0.01\mathrm{mm}\leq s<1\mathrm{mm}\;\\ 0.26\; & \;1\mathrm{mm}\leq s<2\mathrm{mm}\; \end{array}\right.$$

where *τ*_f_ is the bond stress; *s* is the slip; and $f_{c}^{\prime }$ is the cylinder compressive strength of concrete, $f_{c}^{\prime }=29.2\mathrm{MPa}$.

**Figure 9:**
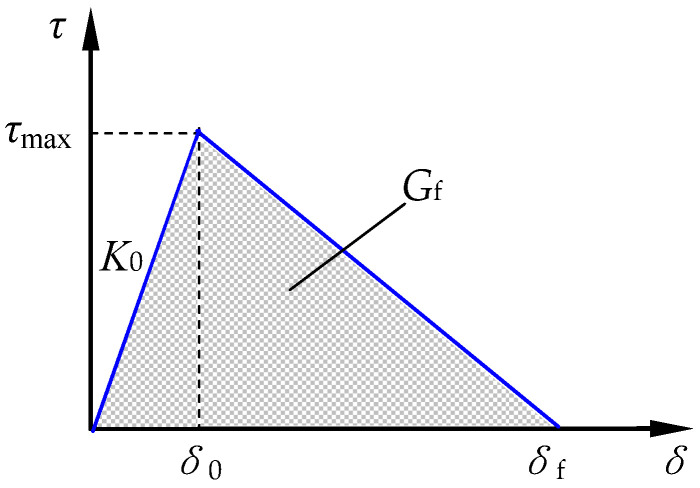
Bond-slip constitutive relation at interface between CFRP and concrete.

The traction-separation-based modeling was used to express the adhesive property of interface between CFRP and concrete. The bond-slip relation of a bilinear model is presented in Figure 9.

Where *δ* is the effective opening displacement; *τ* is the effective traction; *K*_0_ is the initial stiffness; *τ*_max_ is the shear bond strength; *G*_f_ is the fracture energy; and *τ*_max_ and *G*_f_ are calculated by Formulas (12) and (13), respectively [52].

(12) $$\tau_{\max}=11.135{\left(\frac{G_{a}}{t_{a}}\right)}^{0.343}G_{f}$$

(13) $$G_{f}=0.396{\left(\frac{G_{a}}{t_{a}}\right)}^{-0.147}f_{c}{}^{0.124}$$

where *G*${}_{a}$ and *t*_a_ are the shear modulus and thickness of the epoxy; and *f*_c_ is the prism compressive strength of concrete.

### 4.4. Boundary Conditions

The boundary conditions were defined assigning displacements to the reference point of rigid bodies at the left and right of the model. As the left acted as pin support and the right acted as roller support, constraints on translation freedom in X, Y and Z directions and rotation freedom in X and Y directions were added at the left, while constraints on translation freedom in Y and Z directions and the rotation freedom in X and Y directions were added at the right.

## 5. Verification of Test Results with FE Analytical Results

### 5.1. Verification of the Repaired Beam

#### 5.1.1. Strain at Mid-Span Section

**Figure 10:**
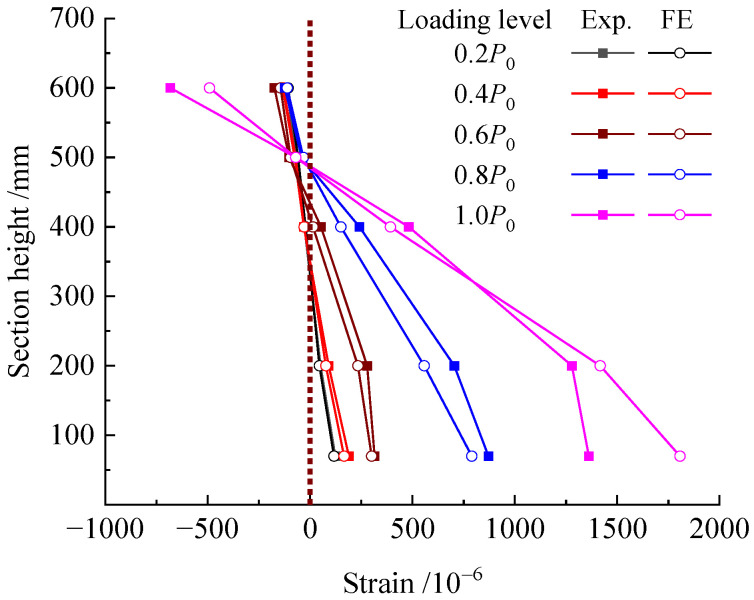
Concrete strain at mid-span section of the repaired beam at different loading level.

The experimental and predicted longitudinal concrete strains at mid-span section are shown in Figure 10; the number before the letter represents the ratio of applied load to *P*_0_. It can be seen that the longitudinal strains of concrete linearly varied at the mid-span section of the repaired beam under loads below the cracking load of 0.6*P*_0_. Agreement presents well between experimental and FE analytical values at this stage. However, the tensile strains of concrete had a larger difference between the values of experiment and the FE analysis when the load was more than the cracking load. This resulted from the cracks due to the tensile stress exceeding the tensile strength of concrete, while the homogeneous tensile strength of concrete was disturbed by the repaired existing cracks in the repaired beam. Generally, the strain maintains a plane at the section of the repaired beam under service load *P*_0_.

#### 5.1.2. Concrete Stress

**Figure 11:**
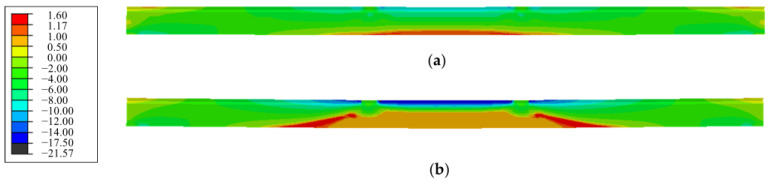
Nephogram of concrete stress along span of the repaired beam under: (**a**) cracking load (0.6*P*_0_); (**b**) service load *P*_0_.

The concrete stresses distribution along span of the repaired beam under the cracking load and the load *P*_0_ at serviceability limit state are shown in Figure 11. Due to the tensile strength of concrete varying from 1.17 MPa to 1.60 MPa based on the detection of the repaired beam, the longitudinal tensile stress was over 1.17 MPa as expressed by RED in the nephogram. As seen in Figure 11a, the longitudinal tensile stress at pure bending sections reached 1.17 MPa, which was the tensile strength of concrete in this area. Thus, the concrete cracks were consistent with the experimental phenomenon of the appearance of vertical macro-cracks in this area, and the predicted load at cracking state was 66 kN (0.6*P*_0_) for the repaired beam.

With the increase in load on the repaired beam, the tensile stress increased in certain tensile zone while the compressive stress increased in the compression zone of the pure bending sections. Due to the unloading effect of the cracked concrete which transferred the tensile stress from the cracked section to the uncracked section, the tensile stress at the BROWN area of the nephogram decreased to be a lower value, while the tensile stress at the web of shear-span near the loading section reached a higher value, which would lead to shear cracks (see Figure 11b). However, due to the better integrity of the web than that of the pure bending segment, the shear crack did not appear at the load *P*_0_. As a result, many flexural cracks appeared in the pure bending sections, which was consistent with the experimental results.

#### 5.1.3. Mid-Span Deflection

**Figure 12:**
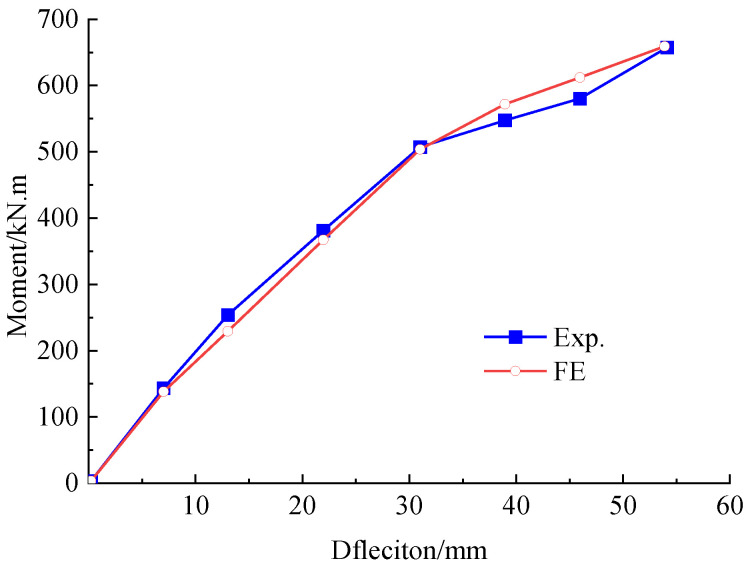
Bending moment versus mid-span deflection curve of the repaired beam under load *P*_0_.

Figure 12 gives the experiment and FE analytical results of the bending moment vs. mid-span deflection to validate the accuracy of the FE model. As can be easily deduced, a good agreement exists between the results obtained from the experiment and the FE analysis. The experiment and calculated mid-span deflection of the repaired beam under the load *P*_0_ were 54.1 mm and 53.9 mm, respectively. This indicates that a differential of 0.35% for the FE analysis, which is feasible to simulate the influence of the initial damage on stiffness by degrading the tensile strength of concrete.

### 5.2. Verification of the Strengthened Beam

#### 5.2.1. Strain at Mid-Span Section

**Figure 13:**
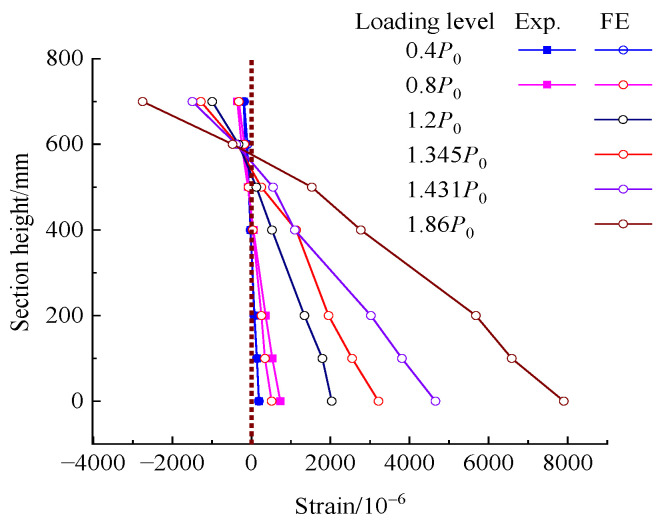
Concrete strain at mid-span section of the strengthened beam at different loading level.

The experimental and FE analytical strains of concrete at the mid-span section are shown in Figure 13. The number before the letter represents the ratio of the applied load to *P*_0_. It can be seen that the longitudinal strains of concrete linearly distribute at the mid-span section, and the results of the experiment agreed well with those of the FE analysis when the load was lower than the cracking load (0.73*P*_0_) for the strengthened beam. From the FE analysis, the strain at the mid-span section basically maintained a plane when the load was over *P*_0_ up until to the ultimate load. Therefore, the assumption of plane strain is adaptable to the bending section of the strengthened beam.

Comparing the concrete strain in Figure 10 and Figure 13, a larger concrete strain was presented on the repaired beam compared to the strengthened beam at the same load level. This can be attributed to the precompression of tensile concrete by the prestressed CFRP plates.

#### 5.2.2. Concrete Stress

**Figure 14:**
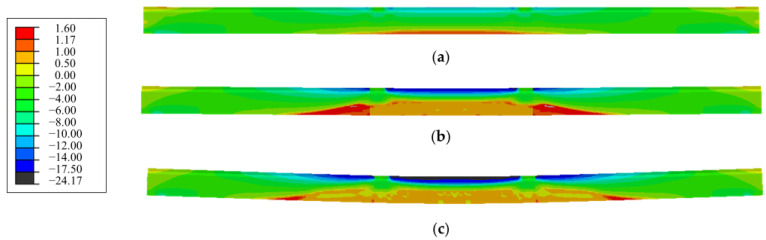
Nephogram of concrete stress along span of the strengthened beam under: (**a**) cracking load (0.73*P*_0_); (**b**) service load *P*_0_; (**c**) ultimate load.

The stress distribution of concrete along the span of the strengthened beam was analyzed under the cracking load, the load *P*_0_ at serviceability limit state, and the ultimate load. The results are shown in Figure 14. When the limit of tensile stress reached the tensile strength of concrete of 1.17 MPa, the first vertical bending crack appeared at the load 80 kN (0.73*P*_0_). This was consistent with the experimental observation. The cracking load increased by 21.2% for the strengthened beam compared to the repaired beam. This verified that the prestressed CFRP plates helped arrest the cracking of the concrete.

**Figure 15:**
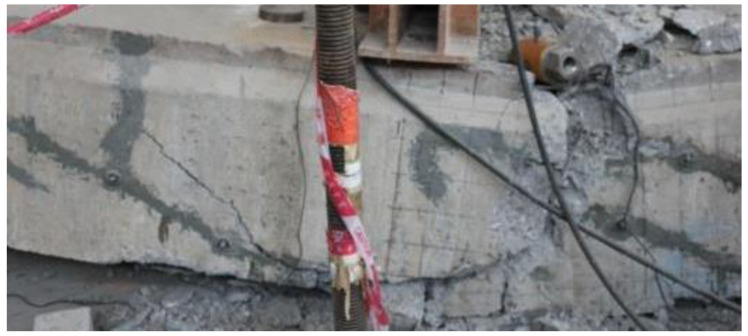
Crushed concrete in compression zone of pure bending segment near loading section.

Similar nephograms existed on the strengthened beams and the repaired beam under the load *P*_0_, except for a lower tensile stress at the shear span of the strengthened beam due to the precompression at the tensile zone by the prestressed CFRP plates. Under the ultimate load, many more vertical cracks at the pure bending segment, accompanied with diagonal cracks in the shear span, appeared on the strengthened beams. The maximum compressive stress at the compression zone reached 24.2 MPa, which was close to the compressive strength 24.7 MPa of concrete. This corresponded to the damage to concrete at the compression zone, as shown in Figure 15. The concrete in compression zone of the pure bending segment near the loading section was crushed in the experiment.

#### 5.2.3. Stress in Prestressed Wires and CFRP Plates

**Figure 16:**
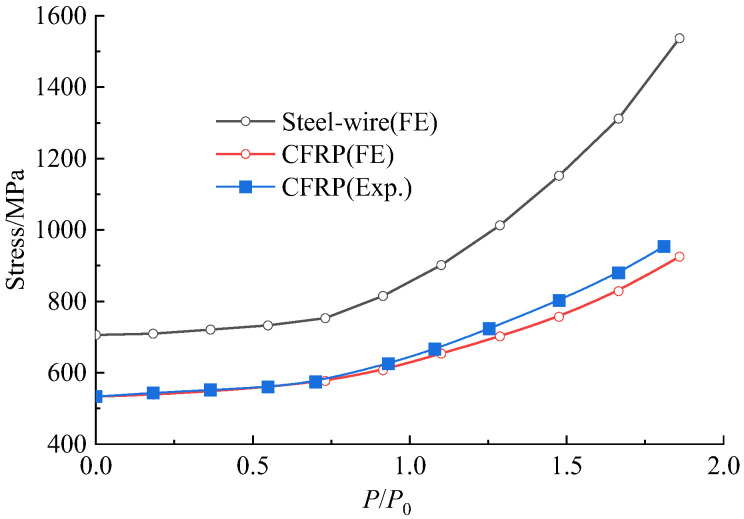
Stresses of prestressed CFRP plates and steel wire changed at different load level.

The comparison of the experiment with the FE analytical results of the tensile stress of prestressed CFRP plates, and the FE analytical results of the tensile stress of prestressed steel wire are presented in Figure 16. The experimental stress of prestressed CFRP plates was taken as the average measured for the three CFRP plates prestressed and bonded on the bottom surface of the repaired beam. Similar stress variation existed for the prestressed CFRP plates. Before the cracking of concrete at the load 0.73*P*_0_, the stress linearly varied with the increase in load. Then, the stress tended to increase quickly, due to the loss of the ability of the cracked concrete to bear tensile stress which was transferred to the CFRP plates and steel wires. The experimental and FE analytical stress of the CFRP plates were 953.7 MPa and 919.5 MPa at the ultimate load. This also demonstrated the validity of the FE model with a difference of 3.6% from the experiment.

Due to the proto beam being taken from an old bridge, the real stress of prestressed steel wire was not available. The stress of the prestressed steel wire of the strengthened beam under its ultimate load was close to the tensile strength of 1600 MPa, using FE analysis. This means that the prestressed steel wire could reach the yield. Meanwhile, the stress of prestressed CFRP plates was lower than the tensile strength of 2400 MPa. This means no brittle fracture of CFRP plates could happen, which was consistent with the experiment where only CFRP plates in some segments unbounded from the concrete surface.

#### 5.2.4. Mid-Span Deflection

**Figure 17:**
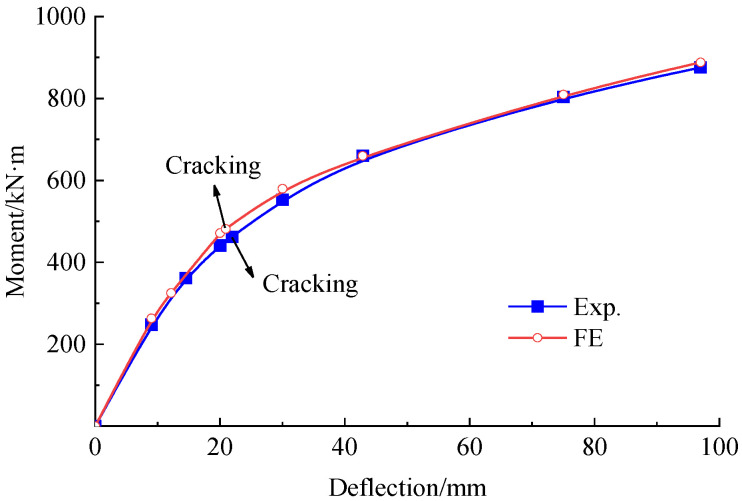
Bending moment vs. mid-span deflection curve of the strengthened beam.

As presented in Figure 17, good agreement is given in the mid-span deflection obtained by the FE analysis and the experiment. The experimental moment at cracking and ultimate states for the strengthened beam were 462 kN·m and 875.7 kN·m, respectively. The corresponded FE analytical values were 481.8 kN·m and 888.4 kN·m. The ultimate moment was close to the target of 883.5 kN·m.

The mid-span deflection of the strengthened beam under the load *P*_0_ was 42.8 mm, which decreased by 20.6% in comparison with that of the repaired beam. This demonstrates that the prestressed CFRP plates effectively improve the stiffness of the strengthened beam, thereby improve the bearing capacity.

## 6. Parametric Analyses of FE Model of Strengthened Beam

In this section, a parametric study is conducted in view of the impacts of the length and amount of prestressed CFRP plates on the loading performance of the strengthened beam in response of the cracking load, ultimate load, and the mid-span deflection.

### 6.1. Effect of the Length of Prestressed CFRP Plates

**Figure 18:**
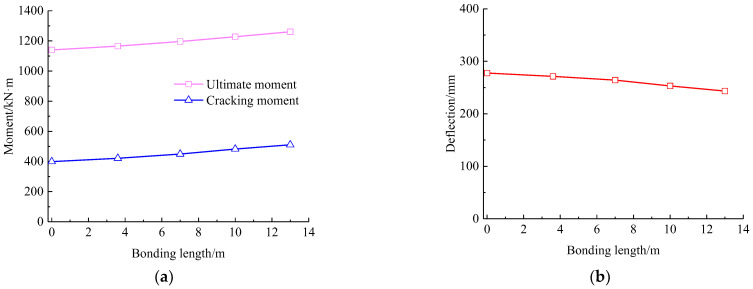
Influence of CFRP bonding length on response of the strengthened beam: (**a**) cracking and ultimate moments; (**b**) ultimate mid-span deflection.

Based on the verification of test results with the FE analysis, the concrete in the compression zone of the strengthened beam was crushed after experiencing large deflections. In this process, of the many cracks appeared in the pure bending segment play a dominate role in the failure of the beam. Therefore, the bonding length of prestressed CFRP plates should be greater than the pure bending segment, i.e., 3.6 m. In this section, the bonding length of prestressed CFRP plates is set to 3.6 m, 7 m, 10 m, and 13 m, with the repaired beam as a reference. Four FE models are built on basis of Section 4 and the analytical results presenting the effect of CFRP bonding length on the cracking moment, the ultimate moment, and corresponded mid-span deflection are shown in Figure 18.

It can be seen that the cracking and ultimate bending moment increase with the increasing bonding length of the prestressed CFRP plate. On the contrary, a decrease presents for the ultimate mid-span deflection. Compared with the repaired beam, the cracking moment and ultimate moment of the strengthened beam increase by 28% and 10.6%, respectively, while the ultimate mid-span deflection reduces by 12.3%. This indicates that a more beneficial effect takes place on the cracking resistance with the increase in bonding length of prestressed CFRP plates. Combined with the concrete stress nephograms, the area of tensile stress over tensile strength of concrete decreases with the increase in the bonding length of prestressed CFRP plates, although the length of tensile area along the span remains unchanged at about 10 m. This reduces the possibility of concrete cracking and confines the extension of cracks in the tensile area. Therefore, the depth of compression zone increases to improve the loading capacity, and the flexural stiffness increases to reduce the mid-span deflection.

In practice, the bonding length of prestressed CFRP plates should be rational to cover the middle segment to confine the possible cracks of concrete. Due to being subjected to moving vehicle loads on bridges, it is better for the simply supported hollow-section beam to be completely strengthened in span by prestressed CFRP plates, except for a certain length near the ends to install anchors and for the convenience of tensioning operation.

### 6.2. Effect of the Amount of Prestressed CFRP Plates

Six FE models were built to investigate the effect of the amount of prestressed CFRP plates on the mechanical properties of the strengthened beam with the repaired beam as a reference. The number of prestressed CFRP plates was set to 3, 6, 9, 12, 15, and 18, respectively. Each plate was the same as the experiment, with a width of 54 mm and a thickness of 1.4 mm. The bonding length was set to 13 m.

**Figure 19:**
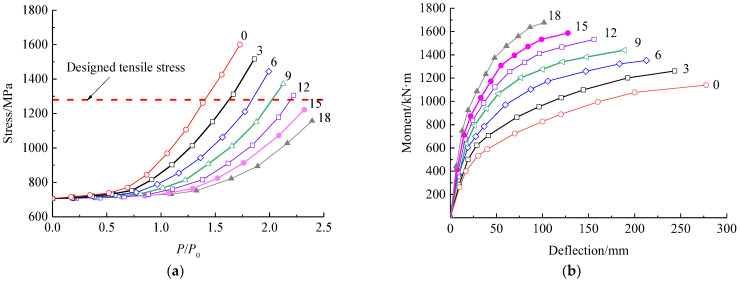
Effect of the amounts of prestressed CFRP plates on: (**a**) the stress of prestressed steel wire; (**b**) mid-span deflection.

Figure 19 presents the tensile stresses of prestressed steel wires and the mid-span deflection changed with the load for the beams with different number of prestressed CFRP plates. From Figure 19a, similar trend of the stress of prestressed steel wire presents with different amount of prestressed CFRP plates. However, the stress of prestressed steel wires slowly increases with the increased number of prestressed CFRP plates. From Figure 19b, the mid-span deflection is obviously lowered with an increase in the amount of prestressed CFRP plates, while the increase in the amount of prestressed CFRP plates leads to a rise in the cracking moment. This improves the serviceability of the strengthened beam at the load *P*_0_ with a smaller deflection due to the enhancement of concrete cracking resistance. With the number of prestressed CFRP plates increased from 3 to 12, the cracking moment increases by 65.6% in total, and each prestressed CFRP plate increases the cracking moment by 7.3%. When the number of prestressed CFRP plates is no less than 12, the crack of concrete can be avoided for the strengthened beam at serviceability limit state.

At the same time, the ultimate bearing capacity can be obviously improved with the failure characterized by the under-reinforced mode transferred to the over-reinforcement mode. When the number of prestressed CFRP plates increases from 3 to 12, the ultimate moment linearly increases by 23.8% in total, while the mid-span deflection decreases by 31.5%. When the number of prestressed CFRP plates is no less than 12, the stress of prestressed steel wire is less than the designed tensile strength of 1280 MPa at the ultimate bearing capacity. This means that the prestressed steel wire cannot yield due to over-reinforcement by prestressed CFRP plates. In this condition, the increase in the ultimate bearing capacity of the strengthened beam is achieved at the expense of deformation ductility, and the ultimate moment.

In practice, to maintain sufficient ductility of the strengthened beam with obviously deflection before failing [9,43], the amount of prestressed CFRP plates should be controlled to let the strengthened beam fail in the under-reinforcement mode.

## 7. Conclusions

In this paper, a hollow-section beam was taken from an old simple support assembly precast concrete bridge which is normally in service for about 25 years. Experimental studies were carried out for the status detection of proto beam, the repair of existed concrete cracks, the strengthening by prestressed CFRP plates, and the loading tests for the repaired and strengthened beams. The FE model is verified by the test results, and used for extending parametric analysis. Conclusions can be drawn as follows:

(1) Although several concrete cracks appeared on the proto beam, the concrete and the prestressed steel wire worked well without obvious degradation and steel erosion. After the concrete cracks were repaired with epoxy adhesive, the degradation coefficient of flexural stiffness was determined as 0.73, based on the experimental study of the repaired beam by four-point bending test.
(2) FE models are built for the analyses of the repaired beam and the strengthened beam, including the concrete strain at mid-span section, the stress of the concrete and the prestressed steel wire, and the stress of prestressed CFRP plates, as well as mid-span deflection. Good agreement between the test and FE analytical results of the loading response demonstrates the validity of the FE model for simulating the strengthened beam with prestressed CFRP plates.
(3) A parametric analysis indicates that the increase in length and amount of prestressed CFRP plates improves the serviceability and the ultimate bearing capacity of the strengthened beam. Additional benefits to improve the flexural stiffness come from the confinements of prestressed CFRP plates to the existed concrete cracks and by increasing the length of prestressed CFRP plates to the newly appeared concrete cracks, and the improvement of concrete cracking resistance by increasing the amount of prestressed CFRP plates.
(4) It should be noticed that with the increased amount of prestressed CFRP plates, the failure mode of the strengthened beam can be transferred from the under-reinforcement to the over-reinforcement. This leads to an expense of deformation ductility of the strengthened beam.

## Figures and Tables

**Table 1:** Material properties of CFRP plates.

*E*_1_ (GPa)	*E*_2_ (GPa)	*N*_u12_	*G*_12_ (MPa)	*G*_13_ (MPa)	*G*_23_ (MPa)
160	9	0.34	4800	4800	4500

## Data Availability

The data presented in this study are available on request from the corresponding author.
